# A Therapeutic Approach for Wound Healing by Using Essential Oils of *Cupressus* and *Juniperus* Species Growing in Turkey

**DOI:** 10.1155/2012/728281

**Published:** 2011-09-18

**Authors:** Ibrahim Tumen, Ipek Süntar, Hikmet Keleş, Esra Küpeli Akkol

**Affiliations:** ^1^Department of Forest Products Chemistry, Faculty of Forestry, Bartin University, 74100 Bartin, Turkey; ^2^Department of Pharmacognosy, Faculty of Pharmacy, Gazi University, Etiler, 06330 Ankara, Turkey; ^3^Department of Pathology, Faculty of Veterinary Medicine, Afyon Kocatepe University, 03200 Afyonkarahisar, Turkey

## Abstract

*Juniperus* and *Cupressus* genera are mainly used as diuretic, stimulant, and antiseptic, for common cold and wound healing in Turkish folk medicine. In the present study, essential oils obtained from cones of *Cupressus* and berries of *Juniperus* were evaluated for their wound healing and anti-inflammatory effects. *In vivo* wound healing activity was evaluated by linear incision and circular excision experimental wound models, assessment of hydroxyproline content, and subsequently histopathological analysis. The healing potential was comparatively assessed with a reference ointment Madecassol. Additionally acetic-acid-induced capillary permeability test was used for the oils' anti-inflammatory activity. The essential oils of *J. oxycedrus* subsp. *oxycedrus* and *J. phoenicea* demonstrated the highest activities, while the rest of the species did not show any significant wound healing effect. The experimental study revealed that *J. oxycedrus* subsp. *oxycedrus* and *J. phoenicea* display remarkable wound healing and anti-inflammatory activities, which support the folkloric use of the plants.

## 1. Introduction

*Juniperus *L. (Cupressaceae) has almost 70 species throughout the world and are mostly distributed in the Northern Hemisphere [1]. The widespread areas extend from Japan and East Asia to Europe, as well as from North and East Africa to North America. In Turkey, the *Juniperus *genus is represented by 10 taxa under seven speciess and has also been used by Anatolian people since ancient times. Fruits of *Juniperus* species are used to produce pekmez (a traditional Turkish fruit concentrate), which is rich in nutritional constituents [2]. Juniper berries are used in Northern European countries to impart sharp and clear flavour to the meat dishes [3, 4]. The coniferous parts and leaves of *Juniperus *are utilized in medicine as an antihelmintic, diuretic, stimulant, and antiseptic and for wound healing. *J. excelsa *is used against tuberculosis and jaundice [5]. The literature survey indicates that the major phenolic constituents in extracts of *Juniperus *species are lignans, coumarins, sesquiterpenes, abietane, labdane and pimarane diterpenes, flavonoids, biflavonols, flavone glycosides, and tannins [1].

The genus *Cupressus* is one of several genera within the family Cupressaceae. Based on genetic and morphological analysis, the *Cupressus* is found in the Cupressoideae subfamily [6]. They are widespread in the northern hemisphere, including western North America, Central America, north-west Africa, the Middle East, the Himalaya, southern China, and north Vietnam. They are evergreen trees or large shrubs, growing to 5–40 m tall. In Turkish folk medicine, fruits of *Cupressus sempervirens* are used to treat common cold and cough [7]. In previous studies, the major constituents have been identified in *Cupressus *species as *α*-pinene and Δ-3-carene [8]. The leaves and fruits of this plant are quite rich in tannins and flavonoids but they are free from alkaloids and low in saponins [9].

The aim of the present study is to evaluate the wound healing activity of the oils from cones of *Cupressus sempervirens *var. *horizontalis* and* C. sempervirens *var. *pyramidalis* and berries of *J. communis*, *J. excelsa*, *J. foetidissima, J. oxycedrus, *and *J. phoenicea* by means of *in vivo* circular excision and linear incision wound models and anti-inflammatory activity.

## 2. Materials and Methods

### 2.1. Plant Material

Berries and cones of 5 different *Juniper* species and 2 different varieties of *Cypress *belonging to Cupressaceae family natively growing in Turkey were chosen as the study material. Specimens were taken from each species as 1 kg weight from their growth sites and stored at −24°C until analysis [10]. Species names, sampling site, local name, collection date, and altitude of all specimens are listed in Table 1.

### 2.2. Hydrodistillation

The essential oils of each sample were obtained by hydrodistillation with a Clevenger apparatus using 250 g (partially crushed) of fresh berries and cones. The essential oils were collected for 3 h and dried over anhydrous sodium sulphate and under refrigeration in a sealed vial until required [11, 12].

### 2.3. Biological Activity Tests

#### 2.3.1. Animals

Male, Sprague-Dawley rats (160–180 g) and Swiss albino mice (20–25 g) were purchased from the animal breeding laboratory of Saki Yenilli (Ankara, Turkey).

The animals were left for 3 days at room conditions for acclimatization. They were maintained on standard pellet diet and water *ad libitum* throughout the experiment. A minimum of six animals were used in each group. Throughout the experiments, animals were processed according to the suggested European ethical guidelines for the care of laboratory animals.

#### 2.3.2. Preparation of Test Samples for Bioassay

Incision and excision wound models were used to evaluate the wound healing activity. For the *in vivo* wound models, test samples were prepared in an ointment base (vehicle) consisting of glycol stearate, 1, 2 propylene glycol, liquid paraffin (3 : 6 : 1) in 1% concentration. 0.5 g of each test ointment was applied topically on the wounded site immediately after wound was created by a surgical blade. 

The animals of the vehicle group were treated with the ointment base only, whereas the animals of the reference drug group were treated with 0.5 g of Madecassol (Bayer, 00001199). Madecassol contains 1% extract of* Centella asiatica*.

For the assessment of anti-inflammatory activity, test samples were given orally to test animals after suspending in a mixture of distilled H_2_O and 1% Tween 80. The control group animals received the same experimental handling as those of the test groups except that the drug treatment was replaced with appropriate volumes of dosing vehicle. Indomethacin (10 mg/kg) in 1% Tween 80 was used as reference drug.

#### 2.3.3. Wound Healing Activity

**Linear Incision Wound Model**

Animals, seven rats in each group, were anaesthetized with 0.15 cc Ketalar [13], and the hairs on the dorsal part of the rats were shaved and cleaned with 70% alcohol. Two 5 cm length linear-paravertebral incisions were made with a sterile blade through the shaved skin at the distance of 1.5 cm from the dorsal midline on each side. Three surgical sutures were placed each 1 cm apart.

The ointments prepared with test samples, the reference drug (Madecassol) or ointment base (glycol stearate: propylene glycol: liquid paraffin (3 : 6 : 1)) were topically applied on the dorsal wounds in each group of animals once daily throughout 9 days. All the sutures were removed on the last day, and tensile strength of previously wounded and treated skin was measured by using a tensiometer (Zwick/Roell Z0.5, Germany) [14, 15].

**Circular Excision Wound Model**

This model was used to monitor wound contraction and wound closure time. Each group of animals (seven animals in each) was anaesthetized by 0.01 cc Ketalar. The back hairs of the mice were depilated by shaving. The circular wound was created on the dorsal interscapular region of each animal by excising the skin with a 5 mm biopsy punch; wounds were left open (Tramontina, Machado, Nogueira Filho Gda, Kim, Vizzioli & Toledo, 2002). Test samples, the reference drug (Madecassol, Bayer) and the vehicle ointments were applied topically once a day till the wound was completely healed. The progressive changes in wound area were monitored by a camera (Fuji, S20 Pro, Japan) every other day. Later on, wound area was evaluated by using AutoCAD program. Wound contraction was calculated as percentage of the reduction in wounded area. A specimen sample of tissue was isolated from the treated skin of each group of mice for the histopathological examination [16].

#### 2.3.4. Hydroxyproline Estimation

Tissues were dried in hot air oven at 60–70°C till consistent weight was achieved. Afterwards, samples were hydrolyzed with 6 N HCl for 4 hours at 130°C. The hydrolyzed samples were adjusted to pH 7 and subjected to chloramine T oxidation. The coloured adduct formed with Ehrlich reagent at 60°C was read at 557 nm [17]. Standard hydroxyproline was also run and values reported as mg/g dry weight of tissue [18].

#### 2.3.5. Histopathology

The skin specimens from each group were collected at the end of the experiment. Samples were fixed in 10% buffered formalin, processed, and blocked with paraffin and then sectioned into 5 micrometer sections and stained with hematoxylin & eosin (HE) and Van Gieson (VG) stains. The tissues were examined by light microscope (Olympus CX41 attached Kameram Digital Image Analyze System) and graded as mild (+), moderate (++), and severe (+++) for epidermal or dermal remodeling. Re-epithelization or ulcer in epidermis, fibroblast proliferation, mononuclear and/or polymorphonuclear cells, neovascularization, and collagen depositions in dermis were analyzed to score the epidermal or dermal remodeling. At the end of the examination, wound healing phases—inflammation, proliferation, and remodeling—were determined for all groups.

#### 2.3.6. Anti-Inflammatory Activity

**Acetic-Acid-Induced Increase in Capillary Permeability**

Effect of the test samples on the increased vascular permeability induced by acetic acid in mice was determined according to Whittle method [19] with some modifications [20]. Each test sample was administered orally to a group of 10 mice in 0.2 mL/20 g body weight. Thirty minutes after the administration, the tail of each animal was injected with 0.1 mL of 4% Evans blue in saline solution (intravenous) and then we waited for 10 min. Then, 0.4 mL of 0.5% (v/v) AcOH was injected intraperitoneally. After 20 min incubation, the mice were killed by dislocation of the neck, and the viscera were exposed and irrigated with distilled water, which was then poured into 10 mL volumetric flasks through glass wool. Each flask was made up to 10 mL with distilled water, 0.1 mL of 0.1 N NaOH solution was added to the flask, and the absorption of the final solution was measured at 590 nm (Beckmann Dual Spectrometer; Beckman, Fullerton, Calif, USA). A mixture of distilled water and 0.5% CMC was given orally to control animals, and they were treated in the same manner as described above.

#### 2.3.7. Statistical Analysis of the Data

The data on percentage anti-inflammatory and wound healing was statistically analyzed using one-way analysis of variance (ANOVA). The values of *P* ≤ 0.05 were considered statistically significant. 

Histopathologic data were considered to be nonparametric; therefore, no statistical tests were performed.

## 3. Results and Discussion

In the present study, wound healing and anti-inflammatory effects of the oils obtained from *Cupressus sempervirens *var. *horizontalis*,* C. sempervirens *var. *pyramidalis J. communis*, *J. excelsa*, *J. foetidissima, J. oxycedrus*, and *J. phoenicea* were evaluated. The oils were mixed with glycol stearate, 1,2 propylene glycol, liquid paraffin (3 : 6 : 1) in 1% concentration for the assessment of the wound healing activity. Linear incision and circular excision experimental wound models were employed. Moreover, skin samples were evaluated histopathologically. The experimental results are given in Tables 2–6.

As shown in Table 2, topical application of the ointment prepared with the oils of aerial parts of *J. oxycedrus* subsp. *oxycedrus *and *J. phoenicea *onto the incised wounds demonstrated the best wound healing activity. On day 10 the tensile strength values were 31.5% and 36.3%, respectively. The rest of the oils did not show any remarkable wound healing activity. 

The contraction values of vehicle, negative control, oils and reference drug treated groups were shown in Table 3. The oils obtained from the berries of *J. oxycedrus* subsp. *oxycedrus *and *J. phoenicea *were found to have wound healing potential, while the vehicle and negative control groups and the other oil ointments showed no statistically significant wound healing activity. The wound contractions were determined as 38.99% and 43.10% for* J. oxycedrus* subsp. *oxycedrus* and 33.28% and 40.74% for *J. phoenicea *when compared to reference drug Madecassol (90.78%–100%).

For the assessment of collagen synthesis hydroxyproline levels of treated tissues were evaluated. As shown in Table 4 significant increase in hydroxyproline content was observed for the tissues treated with the essential oils of *J. oxycedrus *ssp. *oxycedrus *and *J. phoenicea.*

**Figure 1:**
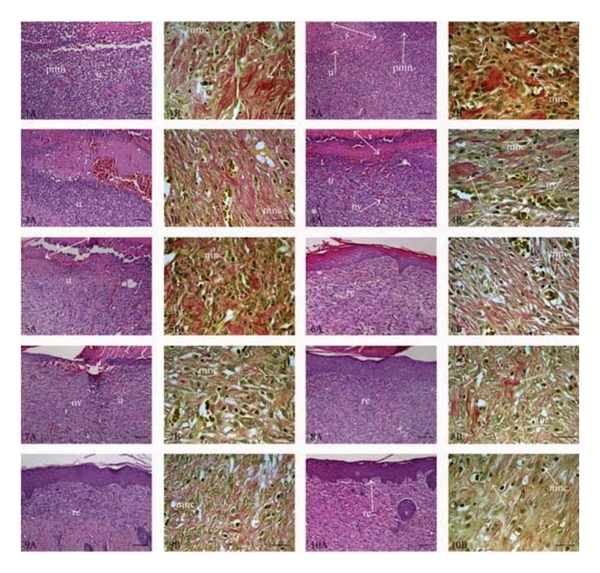
Histopathological view of wound healing and epidermal/dermal remodeling in the vehicle, negative control, oils, and reference ointment Madecassol-administered animals. Skin sections show the hematoxylin & eosin- (HE-) stained epidermis and dermis in A and the dermis stained with Van Gieson (VG) in B. The original magnification was ×100 and the scale bars represent 120 *μ*m for figures in A, and the original magnification was ×400 and the scale bars represent 40 *μ*m for B. Data are representative of 6 animals per group. (1) Vehicle group: 10-day-old wound tissue treated with only vehicle, (2) negative control group: 10-day-old wound tissue, untreated group, (3) *C. sempervirens* var. *horizontalis* group: 10-day-old wound tissue treated with the essential oil of *C. sempervirens* var. *horizontalis*, (4) *C. sempervirens* var. *pyramidalis* group, 10 day old wound tissue treated with essential oil of *C. sempervirens* var. *pyramidalis*, (5) *J. communis* group: 10-day-old wound tissue treated with essential oil of *J. communis*, (6) *J. excelsa* group: 10-day-old wound tissue treated with essential oil of *J. excelsa*, (7) *J. foetidissima* group: 10-day-old wound tissue treated with essential oil of *J. foetidissima*, (8) *J. oxycedrus* group: 10-day-old wound tissue treated with essential oil of *J. oxycedrus*, (9) *J. phoenicea* group: 10-day-old wound tissue treated with essential oil of *J. phoenicea*, and (10) reference group: 10-day-old wound tissue treated with Madecassol. Arrows pointing events during wound healing; s: scab, u: ulcer, re: re-epithelization, f: fibroblast, c: collagen, mnc: mononuclear cells, pmn: polymorphonuclear cells, and nv: neovascularization.

**Figure 2:**
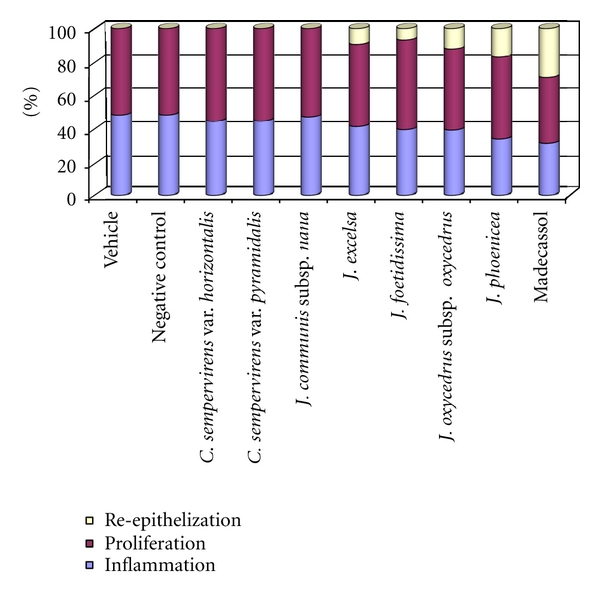
Healing phases of the vehicle, negative control, test ointments, and Madecassol-administered animals.

Phases in wound healing processes (inflammation, proliferation, and remodeling) were observed and within the experimental groups with different degree (Table 5, Figures 1 and 2). The reference drug, essential oils of* J. oxycedrus* subsp. *oxycedrus* and *J. phoenicea *treated groups demonstrated faster remodeling respectively compared to the other groups which showed delayed wound healing processes. As an evidence of delay, wound-associated tissue debris still remained in the dermal tissues. 

The effect of the extracts on the inflammatory phase of wound healing was examined by using the method of Whittle, based on the inhibition of acetic-acid-induced capillary permeability. As shown in Table 6, the inhibitory activity was observed for the oils from *Juniperus oxycedrus *ssp. *oxycedrus *and *J. phoenicea *at the dose of 200 mg/kg with the highest inhibitory values of 33.1% and 27.3%, respectively, while the essential oils from both species showed no significant effect at the dose of 100 mg/kg. On the other hand the rest of the oils did not show any inhibitory activity. 

Previous studies revealed that the essential oils of *Juniperus* and *Cupressus *are rich in *α*-pinene. Particularly *J. phoenicea *possesses the highest *α*-pinene content compared to the other species in comparative phytochemical analysis; *β*-Pinene, sabinene, and limonene are the constituents which are also present in the essential oils of both genera [8, 10]. In a previous study, *α*-pinene was found to have moderate anti-inflammatory effect at 500 mg/kg dose on carrageenan-induced hind paw edema test [21]. Anti-inflammatory activity is essential for wound healing, since a long duration in the inflammatory phase causes a delay in healing process. In order to shorten the healing period as well as for minimal pain and scar, anti-inflammatory activity is required [22]. Presence of *α*-pinene in *Juniperus* species could probably contribute to wound healing activity by providing anti-inflammatory effect. Moreover, limonene is an important monoterpene which has a role in wound healing [23]. In a model of chronic skin inflammation, the researchers have also shown a significant effect of limonene and perillyl alcohol on skin repair and proinflammatory cytokines levels [24]. Furthermore, *J. oxycedrus *ssp. *oxycedrus *and *J. phoenicea *were found to have high flavonoid and phenolic acid content which provide them to show remarkable antioxidant activity [5]. As in many of the diseases, antioxidant activity also helps to promote wound healing process [25].

Collagen is an important extracellular matrix protein which provides strength and integrity to the tissue and plays an important role in haemostasis and epithelisation. High levels of hydroxyproline in regenerated tissue suggest enhanced collagen synthesis. Hence enhanced collagen synthesis by *J. oxycedrus *ssp. *oxycedrus *and *J. phoenicea *may significantly contribute to healing and provide strength to repaired tissue [18].

## 4. Conclusion

According to the experimental results, the oils from berries of *J. oxycedrus *ssp. *oxycedrus *and *J. phoenicea *were found to have better activity on the wound healing compared to the other essential oils and control groups. This might be due to the synergistic effect of the constituents present in the oils. This study provides a scientific evidence for the folkloric utilization of *Juniperus* for wound healing activity.

## Figures and Tables

**Table 1:** Sampling site, local name, date, and altitude of the tree species.

Species	Sampling site	Local name	Collection date	Altitude
*Cupressus sempervirens *var.* horizontalis *	Antalya-Turkey	Dallı Servi	October, 2010	670 m
*Cupressus sempervirens *var.* pyramidalis *	Antalya-Turkey	Piramid Servi	October, 2010	650 m
*Juniperus communis* L. subsp. *nana* Syme.	Keltepe-Karabük	Bodur Ardıç	September, 2010	1,210 m
*Juniperus excelsa* Bieb.	Silifke-Mersin	Boylu Ardıç	October, 2010	220 m
*Juniperus foetidissima* Willd.	Beypazarı-Ankara	Kokulu Ardıç	October, 2010	240 m
*Juniperus oxycedrus* L. subsp. *oxycedrus *	Keltepe-Karabük	Katran Ardıcı	September, 2010	1,200 m
*Juniperus phoenicea* L.	Bodrum-Yokusbasi	Finike Ardıcı	September, 2010	170 m

**Table 2:** Effects of the essential oils from *C. sempervirens* var. *horizontalis*, *C. sempervirens* var. *pyramidalis*, *J. communis*, *J. excelsa*, *J. foetidissima*, *J. oxycedrus,* and *J. phoenicea* on linear incision wound model.

Material	Statistical mean ± SEM (tensile strength %)
Vehicle	13.24 ± 2.25 (9.1)
Negative control	12.14 ± 2.54 —
*Cupressus sempervirens *var.* horizontalis *	14.51 ± 2.17 (9.6)
*Cupressus sempervirens *var.* pyramidalis *	14.27 ± 2.09 (7.8)
*J. communis* subsp. *nana *	13.82 ± 2.04 (4.4)
*Juniperus* *excelsa *	15.27 ± 2.43 (15.3)
*Juniperus* *foetidissima *	14.66 ± 1.86 (10.7)
*Juniperus oxycedrus* subsp. *oxycedrus *	17.41 ± 2.19 (31.5)**
*Juniperus* *phoenicea *	18.05 ± 2.26 (36.3)**
Madecassol	19.87 ± 1.41 (50.1)***

* *P* < 0.05; ** *P* < 0.01; *** *P* < 0.001; SEM: Standard error of the mean.

Percentage of tensile strength values: vehicle group was compared to negative control group; the oils and the reference material were compared to vehicle group.

**Table 3:** Effects of the essential oils from *C. sempervirens *var. *horizontalis, C. sempervirens *var. *pyramidalis, J. communis, J. excelsa, J. foetidissima, J. oxycedrus, *and *J. phoenicea* on circular excision wound model.

Material	Wound area ± SEM (contraction %)						
	0	2	4	6	8	10	12
Vehicle	19.55 ± 2.09	17.98 ± 2.20 (2.55)	16.54 ± 2.11 —	14.89 ± 1.95 —	9.55 ± 1.56 (9.51)	6.31± 1.16 (8.42)	2.97 ± 0.87 (5.41)
Negative Control	19.43 ± 2.15	18.45 ± 2.23	16.42 ± 2.17	14.72 ± 1.91	10.51 ± 1.75	6.89 ± 1.23	3.14 ± 1.49
*C. sempervirens *var.* horizontalis *	19.40 ± 2.13	17.56 ± 1.40 (2.34)	16.02 ± 1.50 (3.14)	14.91 ± 1.62 —	9.57 ± 1.40 —	5.86 ± 1.32 (7.13)	3.56 ± 0.69 —
*C. sempervirens *var.* pyramidalis *	20.03 ± 2.12	17.90 ± 1.91 (0.44)	16.69 ± 1.92 —	14.92 ± 1.60 —	8.84 ± 1.67 (7.43)	7.08 ± 1.12 —	2.59 ± 0.84 (12.79)
*J. communis* subsp. *nana* Syme.	19.55 ± 2.22	17.81 ± 1.81 (5.56)	16.75 ± 1.80 —	15.16 ± 1.87 —	9.57 ± 1.74 —	6.02 ± 0.99 (4.60)	3.04 ± 0.89 —
*J.* *excelsa *	19.50 ± 2.16	14.99 ± 1.78 (16.63)	13.24 ± 2.09 (19.95)	11.26 ± 1.70 (24.38)	7.39 ± 1.58 (22.62)	5.47 ± 1.07 (13.31)	2.44 ± 0.90 (17.85)
*J. foetidissima*	19.96 ± 2.51	16.16 ± 1.89 (10.12)	14.02 ± 1.78 (15.24)	13.49 ± 1.71 (9.40)	9.09 ± 1.54 (4.82)	6.10 ± 1.27 (3.33)	2.49 ± 1.05 (16.16)
*J. oxycedrus* subsp. *oxycedrus *	19.41 ± 2.08	17.51 ± 1.92 (2.61)	16.02 ± 1.70 (6.05)	12.62 ± 1.63 (15.25)	6.03 ± 1.03 (36.86)*	3.85 ± 0.96 (38.99)*	1.69 ± 0.60 (43.10)*
*J. phoenicea*	19.59 ± 2.19	16.46 ± 2.54 (8.45)	14.02 ± 1.96 (15.24)	11.97 ± 1.86 (19.61)	7.44 ± 1.39 (22.09)	4.21 ± 1.28 (33.28)*	1.76 ± 0.61 (40.74)*
Madecassol	19.68 ± 2.03	14.46 ± 1.83 (19.58)	11.77 ± 1.45 (28.84)	8.76 ± 1.21 (41.17)**	4.74 ± 0.89 (50.37)**	2.12 ± 0.31 (66.40)***	0.00 ± 0.00 (100.00)***

* *P* < 0.05; ** *P* < 0.01; *** *P* < 0.001; SEM: standard error of the mean.

Percentage of contraction values: vehicle group was compared to negative control group; the oils and the reference material were compared to vehicle group.

**Table 4:** Effect of topical treatment of test ointments for 7 days on hydroxyproline content.

Material	Hydroxyproline (mg/g) ± SEM
Vehicle	20.21 ± 2.43
Negative Control	19.25 ± 2.67
*C. sempervirens *var.* horizontalis *	18.3 ± 2.41
*C. sempervirens *var.* pyramidalis *	25.9 ± 3.17
*J. communis* subsp. *nana *	24.3 ± 3.52
*J.* *excelsa *	29.5 ± 3.01
*J. foetidissima*	21.8 ± 2.54
*J. oxycedrus* subsp. *oxycedrus *	**50.8** ± 2.44**
*J.* *phoenicea *	**44.3 ** ± 2.81*
Madecassol	**75.6 ** ± 2.95***

* *P* < 0.05; ** *P* < 0.01; *** *P* < 0.001; SEM: standard error of the mean.

**Table 5:** Wound healing processes and healing phases of the vehicle, negative control, test ointments, and Madecassol-administered animals.

Groups	Wound healing processes							
Vehicle	S	U	RE	FP	CD	MNC	PMN	NV
Negative control	++/+++	+++	—	++/+++	++	+++	++/+++	++
*C. sempervirens *var.* horizontalis *	++/+++	++/+++	—	++/+++	++	++	++/+++	++/+++
*C. sempervirens *var.* pyramidalis *	++	++/+++	—	++	++	+/++	++	++
*J. communis* subsp. *nana* Syme.	++/+++	++/+++	—	++/+++	++	+/++	++	+++
*J.* *excelsa *	++/+++	++/+++	—	++	+/++	++	++	+/++
*J. foetidissima*	+/++	+/++	−/+	++	+/++	+/++	+/++	++
*J. oxycedrus* subsp. *oxycedrus *	++	+/++	−/+	++	+/++	+/++	+/++	+/++
*J. phoenicea*	+/++	+/++	−/+	++	+/++	+/++	+/++	++
Madecassol	+/++	−/+	+	++	+/++	+	+/++	+/++

HE- and VG-stained sections were scored as mild (+), moderate (++), and severe (+++) for epidermal and/or dermal remodeling. S: scab, U: ulcer, RE: re-epithelization, FP: fibroblast proliferation, CD: collagen depositions, MNC: mononuclear cells, PMN: polymorphonuclear cells, NV: neovascularization, I: inflammation phase, P: proliferation phase, and R: remodeling phase.

**Table 6:** Inhibitory effect of the essential oils from *C. sempervirens *var. *horizontalis, C. sempervirens *var. *pyramidalis, J. communis, J. excelsa, J. foetidissima, J. oxycedrus, *and *J. phoenicea* on acetic-acid-induced increased capillary permeability.

Material	Dose (mg/kg)	Evans blue concentration (*μ*g/mL) ± SEM	Inhibition (%)
Control		10.69 ± 0.86	
*Cupressus sempervirens* var. *horizontalis *	100	10.40 ± 0.95	2.7
	200	10.12 ± 0.74	5.3
*Cupressus sempervirens* var. *pyramidalis *	100	10.75 ± 0.87	—
	200	11.16 ± 0.91	—
*Juniperus communis *ssp.* nana *	100	10.13 ± 0.79	5.2
	200	10.01 ± 0.38	6.4
*Juniperus excelsa*	100	10.57 ± 0.88	1.1
	200	9.04 ± 0.85	15.4
*Juniperus foetidissima*	100	12.27 ± 0.95	—
	200	11.25 ± 0.99	—
*Juniperus oxycedrus *ssp. *oxycedrus *	100	8.82 ± 0.76	17.5
	200	7.15 ± 0.32	33.1**
*Juniperus phoenicea*	100	9.04 ± 0.89	15.4
	200	7.77 ± 0.44	27.3*
Indomethacin	10	5.28 ± 0.26	50.6***

* *P* < 0.05; ** *P* < 0.01; *** *P* < 0.001 significant from the control; SEM: standard error of the mean.
